# Association among family and domestic violence, sleep disturbance, anxiety, suicidal and self-harm ideation: a chained mediation modeling analysis

**DOI:** 10.3389/fpsyg.2025.1658974

**Published:** 2025-11-14

**Authors:** Chenyu Yan, Yueping Song, Siyuan Liu, Wannian Liang

**Affiliations:** 1 Vanke School of Public Health, Tsinghua University, Beijing, China; 2 Institute for Healthy China, Tsinghua University, Beijing, China; 3 Population Development Studies Center, School of Population and Health, Renmin University of China, Beijing, China; 4 Institute of Health Sciences, School of Population and Health, Renmin University of China, Beijing, China; 5 Big Data and Responsible Artificial Intelligence for National Governance, Renmin University of China, Beijing, China

**Keywords:** family and domestic violence, suicidal ideation, self-harm, sleep disturbance, gender differences

## Abstract

**Introduction:**

Family and domestic violence (FDV) is closely related to suicidal and self-harm ideation (SSI), but the research in this regard is insufficient in China.

**Methods:**

Based on the data from the 2022 Psychology and Behavior Investigation of Chinese Residents, this study used the Generalized Structural Equation Modeling (GSEM) to focus on examining three key aspects: (1) the prevalence of Family and Domestic Violence (FDV) and Suicidal and Self-Harm Ideation (SSI) among the Chinese population; (2) the impact of FDV on SSI, as well as the sequential mediating pathway formed through sleep disturbance and anxiety; (3) the differences in the aforementioned associations across gender and age dimensions.

**Results:**

The research findings indicate that approximately 44% of the participants have experienced FDV, and 25% of the participants have SSI. FDV significantly increases the risk of SSI (1.267, *p* < 0.001). Anxiety mediated the association between FDV and SSI, while sleep disturbance intensified this indirect pathway by increasing anxiety, which in turn elevated SSI risk. By comparing different age groups, it is found that adolescents and the elderly are at a higher risk of developing SSI due to FDV. Gender-specific analysis shows that controlling violence has a significant impact on women, while insulting violence has a stronger impact on men.

**Discussion:**

Based on nationwide residents’ psychological survey data, this study identified the high prevalence of FDV and SSI among the Chinese population as well as their significant association. It also verified the sequential mediating mechanism of sleep disturbance and anxiety between the two, providing new evidence for understanding the harmful pathway of FDV on mental health. Meanwhile, the gender and age differences found in the study suggest that subsequent interventions need to develop targeted strategies based on population characteristics.

## 1 Introduction

Family and domestic violence (FDV) refers to all acts of violence that occur in family relations and intimate partner relationships. It includes physical, sexual, psychological, and other violations committed between partners, parents, and siblings by means of beating, binding, mutilation, restriction of personal freedom, frequent abuse, and intimidation (The National People’s Congress, 2018). Globally, approximately 30% of women and 17% of children have experienced FDV (World Health Organization, 2021a,b; Whitten et al., 2024). In Asia, sociocultural factors such as family disputes, public shaming, and perceptions of marital roles contribute to a higher prevalence of FDV compared to global averages, with 34% of women and 25% of men identified as victims (Shoib et al., 2022; World Health Organization, 2021a,b; Fulu et al., 2013).

Family and domestic violence poses significant threats to both physical and mental health, often culminating in suicide and self-harm, such as cutting, burning, or other self-injurious behaviors (Shoib et al., 2022). Suicide and self-harm are critical health and societal issues, claiming over 0.7 million lives annually and affecting approximately 17% of people over their lifetime (World Health Organization, 2021a,b; Swannell et al., 2014). Suicidal ideation and self-harm thoughts, precursors to attempts, have drawn considerable attention from researchers and practitioners aiming to prevent these outcomes (Mars et al., 2019).

Although prior studies have established a connection between FDV and SSI (Turner and Colburn, 2022; Paul and Ortin, 2019), much of this research originates from high-income Western countries. Considering that 44% of global suicides occur in China, and that low- and middle-income countries such as China bear the greatest burden of suicide and self-harm, it is critical to examine the impact of FDV on SSI within the Chinese context (Knipe et al., 2022; Phillips et al., 2002).

Family and domestic violence victims are also at heightened risk of other health issues, including sleep disturbances and anxiety (Turner et al., 2020; Zola et al., 2021). Severe sleep disturbances can directly or indirectly contribute to obesity, cardiometabolic disease, inflammation, mortality, depression, and anxiety symptoms (Grandner et al., 2012; Cox and Olatunji, 2016). Studies have identified sleep disturbance as a mediator between FDV and mental health outcomes (Lalley-Chareczko et al., 2017; Li et al., 2022), while anxiety has been shown to mediate the relationship between FDV and SSI (Sigfusdottir et al., 2013). However, no research to date has explored the chained mediation effect of sleep disturbance and anxiety linking FDV to SSI. Furthermore, little is known about how gender and age differences influence reactions to different categories of FDV (Sasser et al., 2021).

In China, FDV is prevalent, with approximately 25% of ever-married women experiencing domestic violence from their spouses (Jiang et al., 2013). Most research in China has focused on intimate partner violence within specific groups rather than examining violence perpetrated by all family members across broader populations (Gao et al., 2011). Understanding the prevalence of FDV and its pathways to adverse health outcomes, such as SSI, is essential for designing effective prevention strategies in China. This study, therefore, uses a large representative dataset to: (1) estimate the prevalence of FDV among Chinese individuals aged 12 years and above; (2) examine the association between FDV and SSI; (3) explore the chained mediation mechanisms involving sleep disturbance and anxiety; and (4) analyze gender- and age-related differences in these mechanisms.

## 2 Background

### 2.1 Association between FDV and SSI

Several theoretical frameworks can be utilized to explore the effects of FDV on SSI (O'Connor and Kirtley, 2018; Joiner et al., 2012). The diathesis-stress model posits that FDV, as a stressful experience, interacts with inherited neurobiological vulnerabilities, increasing susceptibility to psychological and personality challenges and thereby heightening SSI risk (Paul and Ortin, 2019). Moreover, SSI is a critical symptom of depression and serves as a key marker for monitoring and mitigating suicidal and self-harming behaviors (Kocalevent et al., 2013). Emotional security theory suggests that FDV undermines emotional security, increasing the likelihood of depressive symptoms, including suicide attempts and self-harm thoughts (Davies and Cummings, 1998).

Research from high-income countries highlights a significant relationship between FDV and SSI. For instance, New Zealand women exposed to physical or sexual violence by intimate partners were more likely to report suicidal thoughts (Gulliver and Fanslow, 2013). Systematic reviews of 52 cross-sectional and longitudinal studies similarly demonstrate a strong association between child maltreatment and suicidal ideation or attempts (Miller et al., 2013). In China, limited research has examined the relationship between FDV and suicidal ideation (Sigfusdottir et al., 2013; Gao et al., 2011). However, the generalizability of these studies is constrained, as they primarily focus on specific subpopulations, such as poorly educated women, and rely on small sample sizes.

### 2.2 Potential mediating effects of sleep disturbance and anxiety in the FDV-SSI relationship

Family and domestic violence has been consistently identified as a significant risk factor for sleep disturbances and anxiety (Turner et al., 2020; Zola et al., 2021; Lind et al., 2015). Research by Turner et al. (2020) and Lind et al. (2015) demonstrates that child neglect and abuse significantly increase the likelihood of sleep problems, such as difficulty falling asleep or frequent awakenings, during adolescence and later life. Similarly, Zola et al. (2021) reported that exposure to FDV often results in heightened anxiety, characterized by persistent tension and worry. The integrated motivational-volitional model, the schematic appraisals model of suicide, and the interpersonal theory of suicide further support that psychological and physiological risk factors, such as sleep disturbances and anxiety, are directly linked to suicidal ideation (O'Connor and Kirtley, 2018; Joiner et al., 2012). Studies conducted in the US, Canada, and Pakistan confirm that both sleep disturbances and anxiety are strong predictors of suicidal and self-harm ideation (Wong et al., 2011; Shahzad et al., 2021).

Considering the complex associations among the above-mentioned variables, potential mediation mechanisms need to be figured out the relationship among them. Anxiety is a key mediator in this relationship. The escape theory of suicide posits that individuals may resort to suicidal attempts or self-harm as a means of escaping overwhelming negative emotions (Palmier-Claus et al., 2012). Supporting this theory, Paul and Ortin (2019) and Sigfusdottir et al. (2013) found that early experiences of neglect, abuse, and family violence indirectly influence suicidal ideation through depressive moods, particularly among adolescents.

Sleep disturbance is another potential mediator in the FDV-SSI relationship. Spilsbury’s (2009) conceptual model suggests that traumatic events, such as FDV, trigger a traumatic response, resulting in sleep disturbances, which can lead to developmental outcomes like anxiety and SSI. Research by Lalley-Chareczko et al. (2017) and Li et al. (2022) highlights the mediating role of sleep disturbances between FDV and mental health outcomes, although the specific effects on SSI remain unclear. Moreover, the strong association between sleep disturbances and anxiety provides theoretical support for chained mediation effects, where FDV leads to sleep disturbances, which in turn heighten anxiety and ultimately result in SSI (Cox and Olatunji, 2016).

The mechanism linking FDV to SSI is complex and multifaceted. To date, no research has comprehensively examined the chained mediating effects of sleep disturbances and anxiety in this context. While some studies have explored pathways between FDV and SSI, these explanations lack clarity, have not been conducted in a Chinese context, and often exclude certain age groups and genders. Further research is required to address these gaps, utilizing diverse and representative populations to improve understanding and inform targeted interventions.

### 2.3 Differences in the associations among different genders and age groups

Both males and females across all age groups face varying risks of exposure to different types of FDV. Adolescence is a particularly vulnerable period marked by rapid physical, psychological, and social developmental changes (Christie and Viner, 2005). Research has consistently shown that maltreatment during adolescence has significant and lasting effects on internalizing behavioral problems, including anxiety, sleep disturbances, and suicidal or self-harm ideation (Paul and Ortin, 2019; Calder et al., 2010). Sleep, which plays a crucial role in adolescents’ mental and physical development, can be disrupted by FDV, further exacerbating anxiety and increasing the risk of suicidal ideation (O’Callaghan et al., 2021).

The elderly are also common victims of FDV, particularly those with health issues such as disabilities (Frazão et al., 2014). However, only a few studies have focused on elderly populations to evaluate the effects of childhood FDV experiences and current maltreatment on suicidal and self-harm ideation. In China, mistreatment significantly increases suicidal ideation rates among the elderly, who often have a higher prevalence of health disorders and greater dependency on family members (Wu et al., 2013; Frazão et al., 2014).

Research on FDV in adults has disproportionately focused on women, often excluding men, despite reports indicating higher exposure to FDV among men in China (Wu et al., 2013). Estimating gender differences in the impacts of FDV on suicidal and self-harm ideation is essential for developing inclusive interventions. Different age groups and genders experience varying degrees of exposure to different forms of FDV, including psychological, physical, and sexual violence (World Health Organization, 2021a,b). Studies in China suggest that victims’ responses to FDV are not uniform (Wu et al., 2013; Gao et al., 2011). To create effective interventions and health services tailored to the diverse needs of FDV victims, further research is needed to explore and compare the effects of FDV on SSI across age groups and genders.

### 2.4 Hypothesis

Based on the theories and findings of the previous studies, we aimed to test the following hypotheses:

*Hypothesis 1*: FDV is associated with higher risks of sleep disturbance, anxiety, and SSI.

*Hypothesis 2*: Sleep disturbance and anxiety sequentially mediate the association between FDV and SSI.

*Hypothesis 3*: The associations among FDV, sleep disturbance, anxiety, and SSI vary across FDV categories, genders, and age groups.

**Figure 1:**
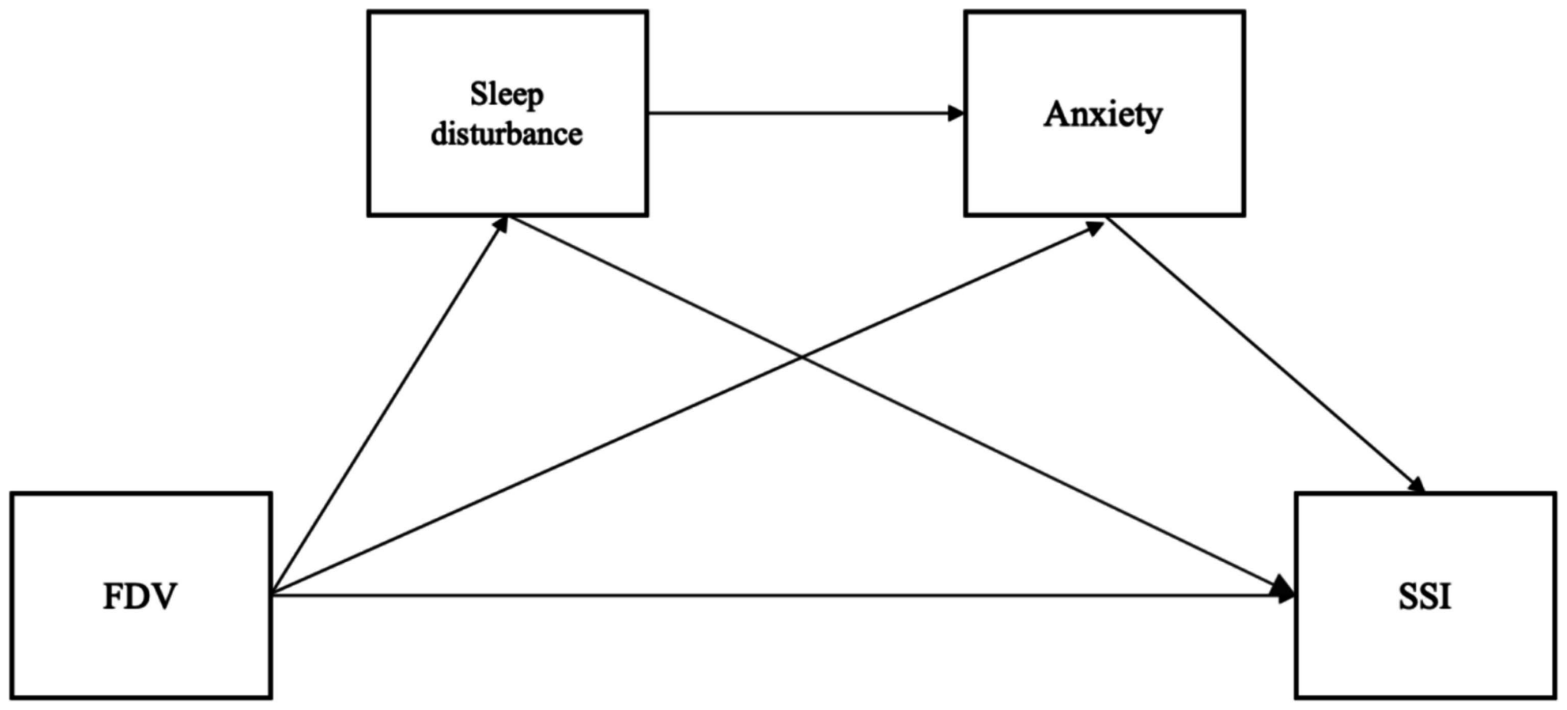
Conceptual framework of the study.

These hypotheses are illustrated in Figure 1.

## 3 Methods

### 3.1 Data source

This study utilized the 2022 Psychology and Behavior Investigation of Chinese Residents (PBICR) dataset for analysis. The PBICR is a cross-sectional survey conducted to investigate the mental health status and health-related behaviors of Chinese residents aged 12 years and older. The survey employed a nationally representative sample, selected using stratified and quota sampling methods. Data were collected from 148 cities, 202 districts and counties, 390 townships/towns/sub-districts, and 780 communities/villages across 23 provinces, 5 autonomous regions, and 4 municipalities directly governed by the central government in China (Fan et al., 2025; Yang et al., 2024). In accordance with the ethical principles of the Ministry of Health’s “Measures for Ethical Review of Biomedical Research Involving Humans (Trial Implementation),” the State Drug Administration’s “Quality Management Standards for Drug Clinical Trials (2003),” “Regulations for Clinical Trials of Medical Devices (2004),” the World Medical Association’s “Declaration of Helsinki” and the “International Ethical Guidelines for Human Biomedical Research,” this dataset passed ethical review (JNUKY-2022-02; 2022-K050). All participants signed the informed consent form. Researchers can apply for de-identified data through the official application channel. For data collection, PBICR 2022 adopted the method of distributing electronic questionnaires.

A total of 21,916 individuals were included in this survey, with a response rate of 71.8% and qualification rate of 96.8%. The qualification rate was calculated after excluding questionnaires with incomplete demographic information, excessive missing items, logically inconsistent answers, or unreasonably short response times.

### 3.2 Variables and measurements

#### 3.2.1 Predictor: family and domestic violence

The FDV was measured by a five-dimensional scale, which included the following aspects: whether one had ever been beaten by family members or boyfriends/girlfriends or been injured with the aid of tools; whether physical contact or sexual behavior had occurred against their will; whether their mobile phones had been checked, their dressing and appearance had been decided, and their interpersonal communication had been restricted; whether they had been compared with others, openly criticized, making them feel embarrassed and unconfident; whether they had not been cared for when they were unwell physically or in a bad mood. These aspects covered Physical Violence, Sexual Violence, Controlling Violence, Criticizing violence, and Emotional Neglect Violence (Duncan et al., 2021).

The responses to each question were designed as “Never,” “Occasionally,” “Sometimes,” “Often,” and “Almost Always.” We adopted two ways to handle the data: (1) Assign values of 0–4 in sequence to obtain the total score for measuring the overall level of FDV (*α* = 0.908 in this sample) (Woodlock, 2017); (2) For each type of violence, assign a value of 0 for the response “Never,” and 1 for other responses, thus generating five dummy variables to measure the extent to which the sample had suffered from the five types of violence.

#### 3.2.2 Outcome: suicidal and self-harm ideation

The SSI was measured by the question “In the past 2 weeks, I have thought about killing myself or self-harming,” which was adapted from previous large-scale population studies (Brener et al., 1995; Kidger et al., 2012). Respondents who answered “Never” were classified as having no tendency of suicide or self-harm, while those who answered “For a few days, on average several days, or almost every day” were classified as having the tendency of suicide or self-harm.

#### 3.2.3 Mediators: sleep disturbance and anxiety

Sleep disturbance was measured by the Brief version of Pittsburgh Sleep Quality Index (B-PSQI) (Sancho-Domingo et al., 2021). The Brief Pittsburgh Sleep Quality Index consists of five questions, including self-evaluation of sleep quality, the time required to fall asleep, the actual sleep time, the degree of difficulty in falling asleep again after waking up in the middle of the night, and the number of days in a week when falling asleep is later than the expected time (Tu et al., 2023). The scoring range of the sleep index is from 0 to 19 points. A global score can be computed and is used in this study (*α* = 0.804 in this sample).

Anxiety was measured by the Generalized Anxiety Disorder Scale (GAD-7). This scale consists of seven items, inquiring about the emotional states of the surveyed subjects in the past 2 weeks and is widely used in anxiety measurement (Plummer et al., 2016). The responses were designed as follows: “Never,” “For a few days,” “Just over half of the days,” and “Nearly every day,” which were assigned values of 0–3 in sequence. The scoring range of the sleep index is from 0 to 21 points. A global score can be computed and is used in this study (α = 0.942 in this sample).

#### 3.2.4 Covariates

Gender is considered to be an important control variable and as such was included in data analyses (female was coded as 0, male was coded as 1). In addition, age, household registration, marital status, years of education, work status, and per capita monthly household income are all included as control variables in the final model. In order to mitigate the heteroscedasticity within the regression, the income is converted into logarithmic form and then incorporated into the regression model.

### 3.3 Statistical analysis

This study adopted the Generalized Structural Equation Model (GSEM), which has gained considerable popularity in the field of mental health research (Zugna et al., 2022). The core outcome variable, SSI, was a binary variable, while sleep disturbance and anxiety were continuous variables. GSEM flexibly adapt to different types of variables (handling binary variables through logit link functions and continuous variables through linear link functions) without the need for distortive data transformation, thus ensuring the accuracy of the analysis. Meanwhile, GSEM enables the simultaneous estimation of direct and indirect effects, limitations of traditional linear models or single regression approaches that struggle to capture complex multivariate and sequential relationships. The robust maximum likelihood estimation (MLR) method implemented in GSEM can tolerate minor deviations from normality, making it well suited for the large sample size of this study and ensuring stable parameter estimation.

The statistical analysis was designed with the dual objective of minimizing the error term and maximizing the explanatory power, thus being based on two consecutive analytical steps. In the initial step, first, sample descriptive statistics were calculated. Categorical variables, such as gender and household registration, were summarized using frequencies and percentages, while continuous variables, such as age, were described using means and standard deviations. To examine bivariate relationships, correlation analyses were conducted between the core predictor variable (FDV), mediating variables (sleep disturbance, anxiety), and the outcome variable (SSI) by both unadjusted models and covariate-adjusted models. Second, a serial multiple mediation model was constructed. The binary outcome variable, SSI, was modeled using a logit link function. The continuous mediating variables, sleep disturbance and anxiety, were modeled using linear link functions. Parameter estimation was performed using maximum likelihood with robust standard errors, which accommodates minor deviations from normality and is well suited for large samples, thereby ensuring stable estimates. The stability of mediation effects was further evaluated using a bootstrap procedure with 1,000 resamples. The direct and indirect mediating effects were evaluated through the GSEM in STATA 18 version software. Additionally, demographic control variables such as age, gender, household registration, and educational attainment were incorporated into the model to enhance its accuracy. All data preparation procedures were carried out in STATA 18 version software.

## 4 Results

### 4.1 Sample characteristics

Table 1 summarizes the means and standard deviations of all continuous variables, the proportions of categorical variables, and the differences between genders and age groups for the variables included in this study. A total of 44.30% of participants reported FDV, and 25.04% of participants reported experiencing SSI. The average FDV Scale score was 2.279.

**Table 1:** Characteristics of the study sample by genders and age groups.

Variable	Category	Complete sample	Gender		*p*-value	Age group			*p*-value
			Female (*n* = 10,958)	Male (*n* = 10,958)		Adolescents (*n* = 3,222)	Youth and middle-aged adults (*n* = 14,571)	Elderly (*n* = 4,123)	
Outcome									
Suicidal and self-harm ideation	Yes	25.04%	23.62%	26.46%	<0.001	28.86%	23.62%	27.07%	<0.001
	No	74.96%	76.38%	73.54%		71.14%	76.38%	72.93%	
Predictor									
Family and Domestic Violence	M ± SD	2.279 ± 3.736	1.995 ± 0.032	2.564 ± 0.039	<0.001	2.768 ± 4.158	2.261 ± 3.671	1.962 ± 3.574	<0.001
	Yes	44.30%	43.38%	45.22%	0.006	48.91%	45.56%	36.24%	<0.001
	No	55.70%	56.62%	54.78%		51.09%	54.44%	63.76%	
Physical violence	Yes	20.32%	17.74%	22.91%	<0.001	23.84%	19.04%	22.12%	<0.001
	No	79.68%	82.26%	77.09%		76.16%	80.96%	77.88%	
Sexual violence	Yes	17.65%	15.00%	20.30%	<0.001	17.50%	17.18%	19.43%	0.004
	No	82.35%	85.00%	79.70%		82.50%	82.82%	80.57%	
Controlling violence	Yes	24.79%	21.63%	27.96%	<0.001	29.52%	24.64%	21.63%	<0.001
	No	75.21%	78.37%	72.04%		70.48%	75.36%	78.37%	
Criticizing violence	Yes	30.63%	27.92%	33.34%	<0.001	39.76%	30.88%	22.60%	<0.001
	No	69.37%	72.08%	66.66%		60.24%	69.12%	77.40%	
Emotional neglect violence	Yes	36.07%	35.36%	36.79%	0.028	36.31%	37.92%	29.37%	<0.001
	No	63.93%	64.64%	63.21%		63.69%	62.08%	70.63%	
Mediators									
Sleep disturbance	M ± SD	4.695 ± 3.423	5.155 ± 0.033	4.776 ± 0.032	<0.001	4.566 ± 3.506	5.084 ± 3.405	4.858 ± 3.393	<0.001
Anxiety	M ± SD	4.719 ± 4.645	4.750 ± 0.043	4.688 ± 0.046	0.331	5.129 ± 5.308	4.744 ± 4.594	4.309 ± 4.217	<0.001
Covariates									
Age	M ± SD	39.428 ± 18.851	39.422 ± 18.811	39.434 ± 18.890	0.481	16.126 ± 1.800	36.302 ± 12.263	68.687 ± 6.316	<0.001
Gender	Female	50.00%				50.00%	50.12%	49.58%	0.826
	Male	50.00%				50.00%	49.88%	50.42%	
Hukou	Rural	46.11%	47.16%	45.05%	0.002	54.41%	40.12%	60.78%	<0.001
	Urban	53.89%	52.84%	54.95%		45.59%	59.88%	39.22%	
Marriage	Currently not Married	43.25%	42.33%	44.17%	0.006	99.16%	38.41%	16.64%	<0.001
	Currently Married	56.75%	57.67%	55.83%		0.81%	61.59%	83.36%	
Work status	Employment	46.59%	44.00%	49.18%	<0.001	1.80%	65.57%	14.50%	<0.001
	Unemployment	23.39%	26.10%	20.68%		0.84%	10.94%	85.01%	
	Student	30.02%	29.91%	30.14%		97.36	23.49	0.49	
Education	M ± SD	12.260 ± 4.559	11.933 ± 4.706	12.186 ± 4.404	<0.001	11.302 ± 3.082	13.424 ± 3.089	7.833 ± 5.186	<0.001
Per capita monthly household income	M ± SD	5197.025 ± 4056.301	4890.765 ± 3816.867	5503.285 ± 4260.585	<0.001	5281.502 ± 4255.215	5507.892 ± 4173.355	4032.379 ± 3171.869	<0.001

*N* = 21,916; Continuous variables were analyzed using *t*-tests and ANOVA, and categorical variables were analyzed using chi-square tests.

Among the five violence categories, emotional neglect violence was the most commonly reported, with 36.07% of participants indicating they had experienced it. This was followed by criticizing violence (30.63%), controlling violence (24.79%), physical violence (20.32%), and forced sexual violence (17.65%). The average scores for the Sleep Disturbance Scale (B-PSQI) and Anxiety Scale (GAD-7) were 4.695 and 4.719, respectively.

Male participants reported higher proportions and scores for SSI and violence exposure compared to females. However, females exhibited higher scores for sleep disturbance and anxiety, with averages of 5.155 and 4.750, respectively, compared to 4.776 and 4.688 for males.

Adolescents (12–17 years old) had the highest prevalence of SSI (28.86%) and FDV (2.768) among all age groups. They also reported the highest proportions of physical violence (23.84%), controlling violence (29.52%), and criticizing violence (39.76%). The elderly (≥60 years old) were most affected by sexual violence (19.43%), while adults (18–59 years old) experienced the highest rates of emotional neglect violence (37.92%). Middle-aged and young adults had the highest average sleep disturbance scores, while adolescents exhibited the highest anxiety scores.

Statistical analyses revealed significant differences between genders for FDV, sleep disturbance, and SSI (*p* < 0.001), except for anxiety. Significant differences were also observed across age groups for all variables (*p* < 0.05). Additionally, covariates such as hukou, marital status, work status, education, and income showed significant differences between gender and age groups (*p* < 0.05).

### 4.2 Correlation among predictors, mediators and outcome

A correlation analysis was conducted to explore the relationships between the independent variable (FDV), mediator variables (sleep disturbance and anxiety), and the dependent variable (SSI) (Table 2). The analysis revealed key associative patterns consistent with Hypothesis 1. FDV was significantly and positively associated with all three core variables of interest, including sleep disturbance (𝛽 = 0.211), anxiety (𝛽 = 0.544), and SSI (OR = 1.267). These results support the hypothesis that FDV is linked to elevated levels of sleep disturbance and anxiety, as well as an increased likelihood of suicidal or self-harm ideation. Among the five subtypes of FDV, physical violence (𝛽 = 0.300), critical violence (𝛽 = 0.656), and emotional neglect violence (𝛽 = 1.353) demonstrated significant correlations with sleep disturbance. Furthermore, all subtypes of FDV, physical violence, sexual violence, controlling violence, critical violence, and emotional neglect violence, were positively associated with anxiety and SSI. Sleep disturbance was positively correlated with both anxiety (𝛽 = 0.491) and SSI (OR = 1.143), while anxiety itself exhibited a strong positive correlation with SSI (OR = 1.388).

**Table 2:** Correlation among family and domestic violence, sleep disturbance, anxiety, and suicidal and self-harm ideation.

	Sleep disturbance		Anxiety		SSI	
	Unadjusted model	Adjusted model	Unadjusted model	Adjusted model	Unadjusted model	Adjusted model
FDV	0.211^***^	0.216^***^	0.544^***^	0.539^***^	1.267^***^	1.266^***^
	[0.198,0.224]	[0.203,0.229]	[0.526,0.562]	[0.521,0.558]	[1.254,1.279]	[1.253,1.278]
Physical violence	0.300^**^	0.335^***^	0.995^***^	0.969^***^	1.879^***^	1.816^***^
	[0.115,0.485]	[0.151,0.520]	[0.755,1.236]	[0.728,1.210]	[1.682,2.100]	[1.624,2.031]
Sexual violence	−0.185	−0.179	0.976^***^	1.099^***^	2.782^***^	2.817^***^
	[−0.382,0.011]	[0.375,0.017]	[0.719,1.233]	[0.842,1.357]	[2.475,3.127]	[2.503,3.170]
Controlling violence	−0.071	−0.019	0.639^***^	0.664^***^	1.376^***^	1.382^***^
	[−0.234,0.093]	[−0.182,0.143]	[0.428,0.851]	[0.454,0.874]	[1.233,1.534]	[1.239,1.542]
Criticizing violence	0.656^***^	0.683^***^	1.297^***^	1.162^***^	1.462^***^	1.449^***^
	[0.507,0.806]	[0.534,0.832]	[1.109,1.486]	[0.973,1.350]	[1.320,1.620]	[1.306,1.608]
Emotional neglect violence	1.353^***^	1.286^***^	1.339^***^	1.333^***^	1.086^*^	1.107^*^
	[1.219,1.486]	[1.153,1.418]	[1.175,1.504]	[1.169,1.497]	[1.009,1.196]	[1.005,1.219]
Sleep disturbance			0.491^***^	0.487^***^	1.143^***^	1.145^***^
			[0.472,0.510]	[0.468,0.506]	[1.133,1.153]	[1.135,1.156]
Anxiety	0.267^***^	0.265^***^			1.388^***^	1.391^***^
	[0.256,0.277]	[0.254,0.276]			[1.372,1.404]	[1.375,1.407]

When taking sleep disturbance and anxiety as the dependent variables, multiple linear regression was established. When taking suicidal and self-harm tendencies as the dependent variable, Logistic regression was established. The covariates have been controlled in the adjusted model. The regression coefficients under robust standard errors were all presented, with the 95% confidence intervals shown in parentheses. *p < 0.05, **p < 0.01, ***p < 0.001.

We further examined the moderating effects of education and income. The results indicated that higher educational attainment significantly strengthened the negative association between emotional neglect and SSI (Supplementary Figure S1). Individuals with higher education levels may be more sensitive to emotional neglect, as education enhances their awareness and understanding of emotional abuse. In contrast, higher income levels significantly attenuated the adverse effects of critical and controlling violence on SSI tendencies (Supplementary Figures S2–S4). These findings underscore the importance of economic support, suggesting that improving household income can effectively buffer the detrimental mental health impacts of FDV.

### 4.3 Chained moderated mediation modeling of SSI

**Figure 2:**
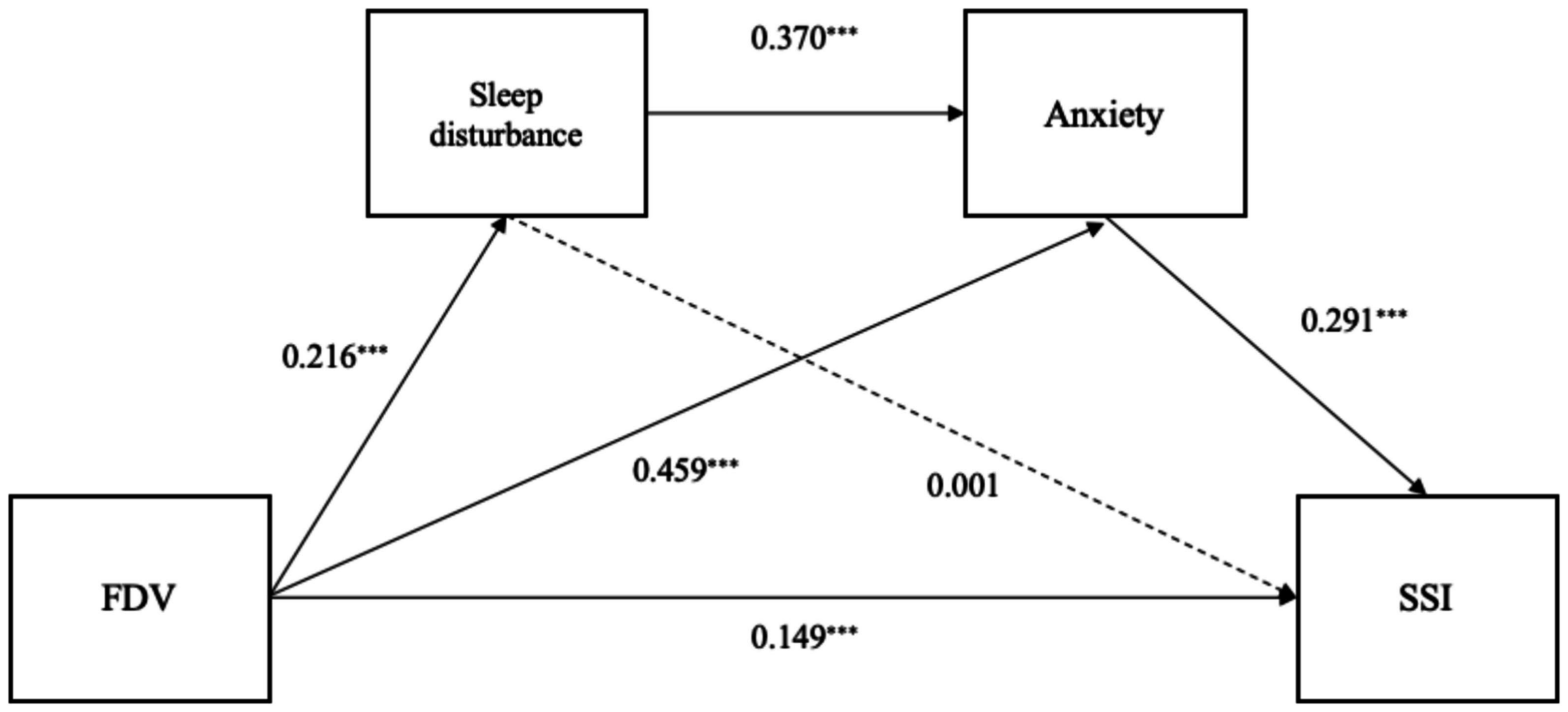
Chained mediation modeling of the associations among Family and Domestic Violence, Sleep Disturbance, Anxiety, and Suicidal and self-harm ideation. AUC = 0.801, CFI = 0.916, RMSEA = 0.045. Solid lines represent that the coefficients are significant (α = 0.05), while dashed lines indicate non-significance. Only the significant coefficients are presented in the diagram. *p < 0.05, **p < 0.01, ***p < 0.001.

The results confirmed the hypothesized sequential mediation model, with FDV scores and the five FDV categories as independent variables, sleep disturbance as the first mediator, anxiety as the second mediator, and SSI as the dependent variable. The standardized path coefficients are illustrated in Figure 2, while the Generalized Structural Equation Modeling (GSEM) results are presented in Table 3 and Supplementary Figure S1. To control the confounding effects of different types of violence, we included all violence types in all models.

**Table 3:** Standardized direct, indirect, and total effects of family and domestic violence on suicidal and self-harm ideation.

	GSEM1			GSEM2		
	Sleep disturbance	Anxiety	SSI	Sleep disturbance	Anxiety	SSI
FDV	0.216^***^	0.459^***^	0.149^***^			
	[0.204,0.228]	[0.445,0.474]	[0.139,0.159]			
Physical violence				0.339^***^	0.841^***^	0.483^***^
				[0.166,0.512]	[0.629,1.053]	[0.352,0.615]
Sexual violence				−0.169	1.163^***^	1.029^***^
				[−0.355,0.017]	[0.935,1.391]	[0.890,1.168]
Controlling violence				−0.015	0.669^***^	0.216^***^
				[−0.175,0.145]	[0.474,0.865]	[0.089,0.344]
Criticizing violence				0.677^***^	0.906^***^	0.124^*^
				[0.531,0.823]	[0.726,1.085]	[0.003,0.245]
Emotional neglect violence				1.282^***^	0.848^***^	−0.296
				[1.155,1.409]	[0.691,1.006]	[−0.409,0.184]
Sleep disturbance		0.370^***^	0.001		0.378^***^	0.011
		[0.354,0.386]	[−0.011,0.013]		[0.362,0.394]	[−0.001,0.023]
Anxiety			0.291^***^			0.294^***^
			[0.281,0.302]			[0.283,0.304]

The covariates have been controlled. The regression coefficients under robust standard errors were all presented, with the 95% confidence intervals shown in parentheses. *p < 0.05, ***p < 0.001.

The analysis revealed a significant sequential indirect effect of FDV scores on SSI, with sleep disturbance as the initial mediator and anxiety as the subsequent mediator. The direct effect of FDV on SSI accounted for 48.9% of the total effect (𝛽=0.149,95%CI = [0.099,0.233]), while the sequential mediation pathway—where FDV was positively associated with sleep disturbance, sleep disturbance was positively associated with anxiety, and anxiety was positively associated with SSI—accounted for 7.8% of the total effect (𝛽=0.024, 95%CI = [0.019, 0.037]). For detailed information, please refer to Supplementary Table S1. These findings provide strong evidence supporting the partial mediation hypothesis (Hypothesis 2).

### 4.4 Gender and age group differences in the associations between FDV and SSI

**Figure 3:**
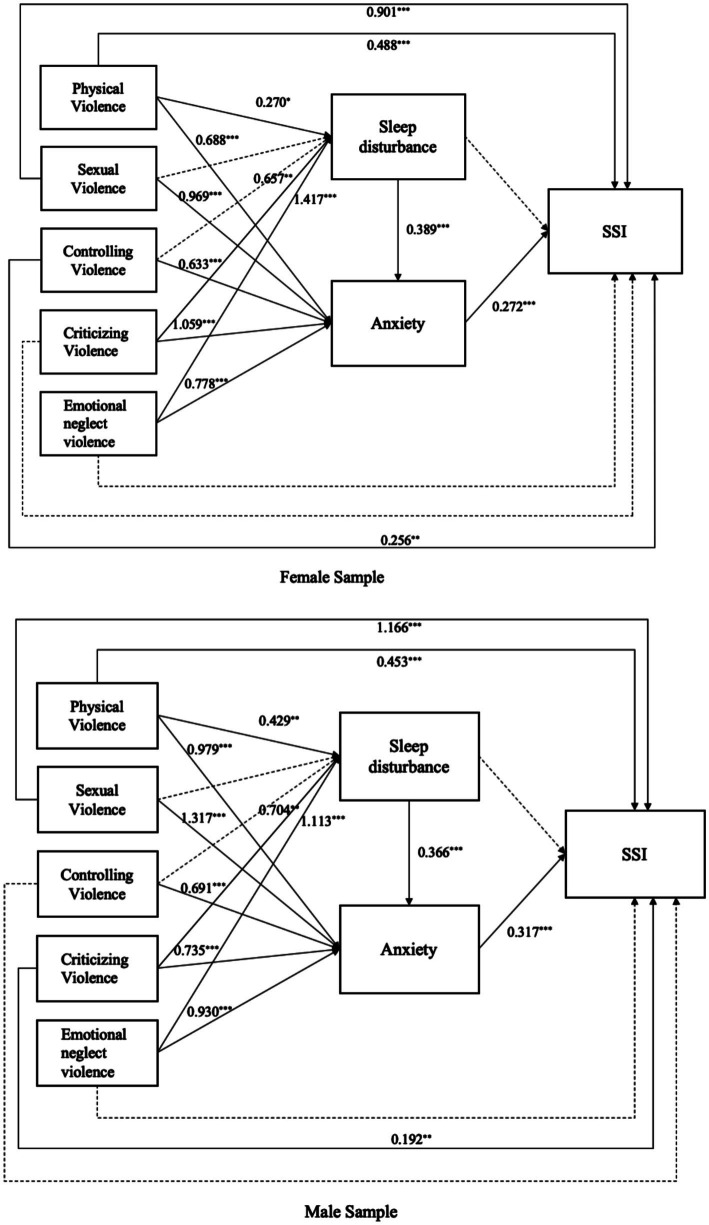
Chained mediation modeling of the associations among Family and Domestic Violence, Sleep Disturbance, Anxiety, and Suicidal and self-harm ideation by genders. Female AUC = 0.876, CFI = 0.919, RMSEA = 0.048, Male AUC = 0.882, CFI = 0.923, RMSEA = 0.048. Solid lines represent that the coefficients are significant (α = 0.05), while dashed lines indicate non-significance. Only the significant coefficients are presented in the diagram. *p < 0.05, **p < 0.01, ***p < 0.001.

Figure 3 illustrates the standardized paths of the five FDV subtypes on SSI, stratified by gender. Both physical violence and sexual violence exert direct effects on SSI in male and female participants, with physical violence showing path coefficients of 𝛽 = 0.488 for females and 𝛽 = 0.453 for males, and sexual violence exhibiting stronger effects (𝛽 = 0.901 for females and 𝛽 = 1.166 for males).

Gender-specific patterns emerged for the other FDV subtypes. Controlling violence demonstrated a direct effect on SSI for females (𝛽 = 0.256), while criticizing violence had a direct effect on SSI for males (𝛽 = 0.192). Sequential mediation effects involving sleep disturbance as the first mediator and anxiety as the second mediator were significant for both male and female participants in the case of physical violence. However, gender differences were evident in other paths: among females, controlling violence was associated with a significant mediation effect by anxiety (𝛽 = 0.172, 95%CI = [0.122, 0.301]), while for males, a sequential mediation effect was observed in the path where criticizing violence served as the independent variable (𝛽 = 0.085, 95%CI = [0.043, 0.198]).

**Figure 4:**
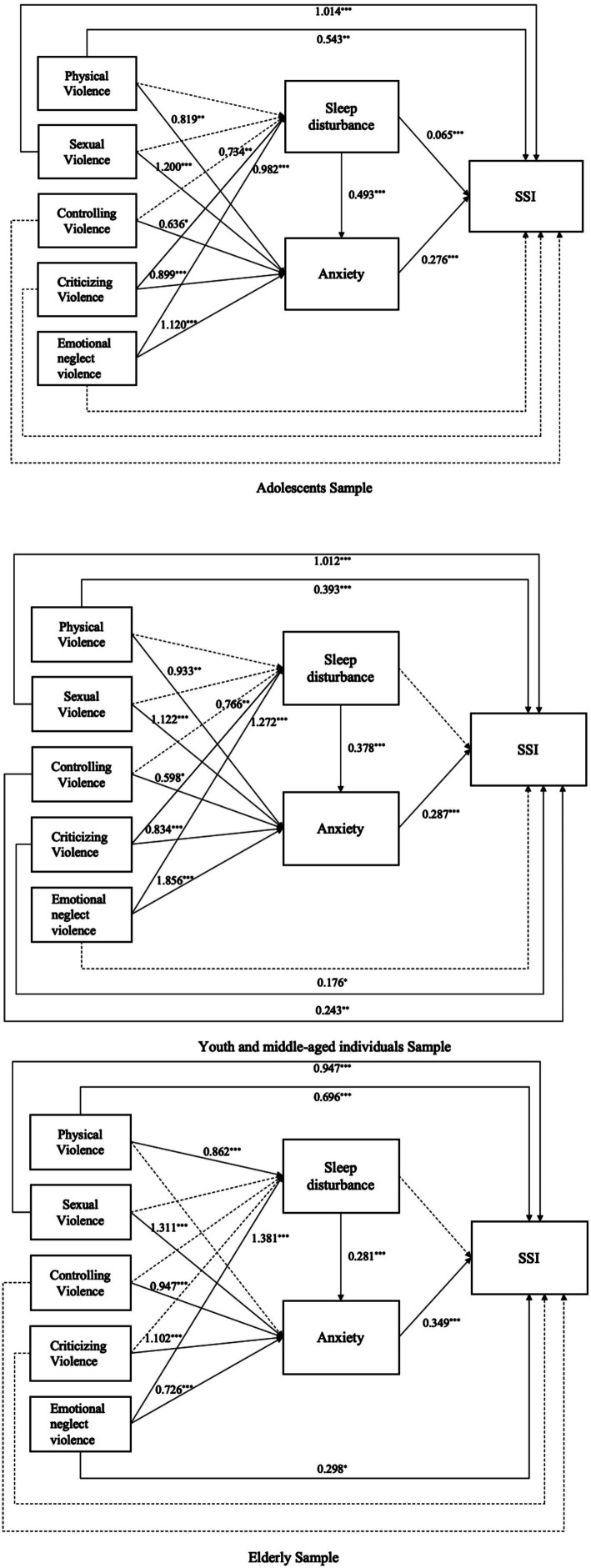
Chained mediation modeling of the associations among Family and Domestic Violence, Sleep Disturbance, Anxiety, and Suicidal and self-harm ideation by age groups. Adolescents AUC = 0.807, CFI = 0.922, RMSEA = 0.048. Youth and middle-aged AUC = 0.871, CFI = 0.923, RMSEA = 0.048. Elderly AUC = 0.883, CFI = 0.921, RMSEA = 0.067. Solid lines represent that the coefficients are significant (α = 0.05), while dashed lines indicate non-significance. Only the significant coefficients are presented in the diagram. *p < 0.05, **p < 0.01, ***p < 0.001.

Figure 4 illustrates the standardized path diagrams stratified by age groups, revealing significant age-specific patterns in the relationship between FDV and SSI. Among adolescents, physical violence (𝛽 = 0.543) and sexual violence (𝛽 = 1.014) exhibit direct effects on SSI, with no evidence of a serial mediating effect through sleep disturbance and anxiety. Compared to youth, middle-aged, and elderly groups, adolescents show a stronger positive correlation between sleep disturbance and SSI.

For the youth and middle-aged group, physical violence (𝛽 = 0.393), sexual violence (𝛽 = 1.012), controlling violence (𝛽 = 0.243), and criticizing violence (𝛽 = 0.176) directly influence SSI. Additionally, sleep disturbance and anxiety demonstrate a significant sequential mediating effect in the pathway from criticizing violence to SSI (𝛽 = 0.083, 95%CI = [0.041, 0.209]).

In the elderly group, physical violence (𝛽 = 0.696), sexual violence (𝛽 = 0.947), and emotional neglect violence (𝛽 = 0.298) directly impact SSI. Sleep disturbance and anxiety serve as serial mediators in the relationships between both physical violence (𝛽 = 0.085, 95%CI = [0.029, 0.165]) and emotional neglect violence (𝛽 = 0.071, 95%CI = [0.068, 0.133]) with SSI. For detailed information, please refer to Supplementary Table S2. These findings highlight significant gender- and age-specific differences in the effects of the five types of FDV on SSI, supporting Hypothesis 3.

## 5 Discussion

In this study, we investigated the effects and mechanisms of FDV on SSI among individuals aged 12 years and older using the GSEM method. Our findings revealed that above 40% of participants had experienced FDV, and one in four individuals reported SSI in China. All five categories of FDV, physical violence, sexual violence, controlling violence, criticizing violence, and emotional neglect violence, were significantly and positively associated with SSI. A substantial portion of these associations was mediated by anxiety. Furthermore, sleep disturbance arising from FDV contributed to increased anxiety, which sequentially led to SSI. The pathways linking FDV to SSI varied significantly across gender and age groups.

This study is the first to estimate the prevalence rates of five types of violence perpetrated by both family members and intimate partners among Chinese adolescents and adults. Compared with the 2010 Chinese National Women Survey, which reported that 28.83% of married women had experienced domestic violence (Song et al., 2020), our study revealed a higher proportion (44.30%) of FDV in the population. This difference is partly attributable to the broader scope of our FDV measurement, which included additional types and sources of violence (e.g., sexual violence encompassing physical contact or sexual behavior). Notably, our findings show that more males and adolescents were FDV victims compared to females and adults. From a traditional gendered cultural perspective, women may report relatively lower FDV due to patriarchal beliefs and the virtue of tolerance, which dictate that women should not question or challenge the authority of their parents or partners (Breckenridge et al., 2019). Adolescents reported higher FDV exposure due to the prevalent authoritarian parenting style in China, characterized by corporal punishment and mind control (Liu et al., 2022).

Consistent with previous studies (Miller et al., 2013; Paul and Ortin, 2019), our results confirmed the positive association between FDV and SSI among the Chinese population, supporting Hypothesis 1. Most of the explanatory power was retained in the direct effects of FDV on SSI. Beyond direct effects, FDV and its five subtypes indirectly impacted SSI through anxiety, supporting part of Hypothesis 2. These findings align with the interpersonal and escape theories of suicide, which propose that FDV, as a significant stressor, exacerbates anxiety and feelings of burdensomeness, ultimately leading to suicidal and self-harm ideation (Joiner et al., 2012; Palmier-Claus et al., 2012). While prior research identified anxiety’s mediating role between FDV and SSI primarily in adolescents (Paul and Ortin, 2019; Sigfusdottir et al., 2013), this study demonstrated similar effects among adults. In addition, although sleep disturbance was significantly associated with SSI among adolescents consistent with prior findings (e.g., Wong et al., 2011; O’Callaghan et al., 2021), our full-sample analysis did not detect a significant direct effect. This divergence may be partly explained by variations in in measurement instruments, population characteristics, and cultural context. Nevertheless, a significant indirect pathway was observed, in which sleep disturbance heightened anxiety, thereby increasing the risk of SSI.

No prior study has examined the chained mediation effects of sleep disturbance and anxiety on the association between FDV and SSI. While indirect effect size was modest, the findings of this study support the hypothesized sequential mediation model, demonstrating that FDV influences SSI indirectly through sleep disturbance as the first mediator and anxiety as the subsequent mediator. While previous research has explored the correlations between violence, sleep disturbance, anxiety, and SSI (Neyazi et al., 2024; Li et al., 2022), and studies like Lalley-Chareczko et al. (2017) have identified sleep disturbance as a mediator in the relationship between intimate partner violence and mental health, the current study expands the theoretical framework by illustrating how FDV contributes to SSI through these sequential pathways. Compromised sleep quality, often a consequence of biopsychosocial stressors like FDV, can lead to daily dysfunction, impaired memory, impatience, and reduced concentration (Buysse et al., 2011). These symptoms cumulatively exacerbate anxiety, thereby heightening the risk of SSI (Neyazi et al., 2024).

Gender differences in the association between FDV types and SSI are particularly noteworthy. Physical and sexual violence had significant direct effects on SSI in both males and females, while not all psychological violence exhibited direct impacts. Controlling violence had a direct effect on SSI only in females. From a feminist poststructuralist perspective, controlling FDV (e.g., monitoring mobile phone usage, restricting dress and interpersonal communication) reflects practices of ownership, surveillance, and identity control by parents or partners (Towns and Scott, 2013). These findings align with objectification theory, which posits that women in patriarchal contexts are socialized to internalize external surveillance and control, thereby experiencing heightened self-monitoring, body dissatisfaction, and anxiety (Fredrickson and Roberts, 1997). This control undermines autonomy development—a core psychological need in self-determination theory—leading to increased stress and vulnerability to SSI (Ryan and Deci, 2000). Such dynamics, particularly within patriarchal cultures, disproportionately affect females, exacerbating their sleep disturbances, anxiety, and subsequent SSI tendencies (Brown, 2014).

Conversely, criticizing violence, characterized by comparison to others, public criticism, and inducing embarrassment or low confidence, significantly affected SSI in males. This pattern can be interpreted through the framework of masculinity threat theory, which posits that men in gender-privileged cultures often derive self-worth from public recognition, achievement, and “face.” Psychological violence that humiliates or undermines competence may activate loss-of-face experiences and social defeat schemas, which are well-established risk factors for depression and suicidality (Gilbert, 2000). In traditional Chinese culture, where men hold a gender-privileged status, psychological demeaning violence may undermine self-esteem and provoke feelings of “loss of face,” making males particularly vulnerable to SSI under these conditions (Wang and Pan, 2024; Jiang et al., 2013).

Age-specific variations in the effects of FDV on SSI were also observed. Adolescents exhibited higher direct effects of physical and sexual violence on SSI, while psychological violence influenced SSI indirectly through sleep disturbance and anxiety. Notably, adolescents displayed a stronger positive correlation between sleep disturbance and SSI compared to other age groups. This aligns with previous studies emphasizing sleep’s critical role in adolescent brain development, neural restoration, and physiological maintenance; poor sleep quality can significantly heighten self-harm risk (Cox and Olatunji, 2016; Wong et al., 2011). From a developmental psychology perspective, adolescence is a stage characterized by identity formation and heightened sensitivity to social stressors (Erikson, 1968). Exposure to FDV during this critical period disrupts autonomy development and emotional regulation, thereby amplifying vulnerability to sleep disturbance, anxiety, and SSI.

Among the elderly, emotional neglect violence had significant direct impacts on SSI. Declines in physical function and social networks in later life, coupled with a reliance on family for support, make elderly individuals more susceptible to abuse and its psychological consequences (Fiske and O'Riley, 2016). According to social gerontology theories, such as socioemotional selectivity theory (Carstensen, 1992), older adults place greater emphasis on emotionally meaningful relationships, making them especially vulnerable to the harm caused by neglect or emotional deprivation. Furthermore, the “double jeopardy” framework in aging research suggests that health decline combined with loss of social roles increases psychological frailty, so that unmet emotional needs and neglect from close family members may directly trigger despair and SSI. Emotional neglect violence, in particular, reflects unmet emotional needs, contributing to SSI in this age group.

This study provides robust evidence for individuals, families, policymakers, and society to take proactive steps in addressing FDV and its implications for SSI. First, the high prevalence of FDV, exceeding global averages, and its significant impact on SSI highlight the urgency of public education campaigns addressing all categories of FDV and their consequences. Stronger laws and regulations are needed to protect FDV victims across all demographics in China (World Health Organization, 2021a,b; Whitten et al., 2024). Second, given the mediating roles of sleep disturbance and anxiety in the FDV-SSI relationship, routine screenings for these conditions in communities, schools, and hospitals are essential. High-risk individuals should have access to psychological counseling services that address anxiety linked to sleep disturbances. Third, gender-specific interventions are necessary, with strategies targeting controlling FDV in females and criticizing FDV in males. Fourth, prevention measures must be tailored to different age groups, accounting for intergenerational relationships in adolescents, intimate relationships in adults, and neglect and emotional needs in the elderly.

This study has several strengths. It utilized a large, nationally representative dataset, encompassing both genders and multiple age groups, enabling the generalization of findings to the broader Chinese population. By analyzing physical, sexual, and psychological violence as well as overall FDV scores, this study captured the compounded risks of multiple victimizations, which often confer greater adverse outcomes than single subtypes (Sharratt et al., 2023). Additionally, the chained mediation and heterogeneity analyses provide valuable data for future etiological and intervention studies focused on reducing SSI.

We also recognized some limitations of this study. First, due to the cross-sectional nature of the dataset, causal inferences cannot be drawn. This inherent limitation highlights the need for future longitudinal studies to track changes in FDV, sleep disturbance, anxiety, and SSI over time, thereby validating the observed associations and clarifying potential causal directions. Second, both FDV exposure and SSI outcomes were assessed using self-report scales. Given that data collection occurred during the COVID-19 pandemic, social desirability bias may have influenced responses, as participants might have underreported sensitive experiences to conform to social norms, potentially leading to an underestimation of the observed associations. Third, the current study did not capture detailed information on the frequency or severity of FDV and SSI, nor did it distinguish between suicidal ideation and self-harm ideation. Prior evidence suggests that females are more likely to experience frequent and severe FDV compared to males; thus, future research should explicitly incorporate these dimensions to further explore gender-specific exposure patterns and their differential impacts on mental health outcomes. Future studies should also examine differences in SSI frequency and distinguish between suicidal ideation and non-suicidal self-injury and to refine understanding of their distinct and shared pathways. Finally, although the chained mediation pathway via sleep disturbance and anxiety revealed important insights into underlying mechanisms, the indirect effect size was modest. Future research should examine additional mediators—such as depression, social support, or coping resources—to capture a fuller range of mechanisms linking FDV to SSI.

## 6 Conclusion

This study advances the existing theoretical framework by confirming the sequential mediation roles of sleep disturbance and anxiety in the relationship between FDV and SSI. While physical and sexual violence significantly affected both genders and all age groups, the impact of psychological violence varied. Females’ SSI was particularly sensitive to controlling violence, whereas males’ SSI was more affected by criticizing and insulting violence. Additionally, adolescents and the elderly were more vulnerable to SSI induced by FDV compared to youth and young adults. Given the distinct mechanisms linking FDV to SSI across subgroups, we recommend routine screening for FDV, sleep disturbance, and anxiety. Targeted interventions tailored to specific demographic and psychological needs are crucial for mitigating SSI risk and promoting mental health.

## Data Availability

The data analyzed in this study is subject to the following licenses/restrictions: this study has received data support from the PBICR project team, and the data sources are legal and valid. PBICR has not opened a public application channel. For a detailed introduction to this dataset, please visit: https://m.x-mol.com/groups/pbicr/research. If you need to apply for the use of these data, you can obtain it by contacting the email address bjmuwuyibo@outlook.com. Requests to access these datasets should be directed to Yibo Wu, bjmuwuyibo@outlook.com.
