# Supplementary material for: Association among family and domestic violence, sleep disturbance, anxiety, suicidal and self-harm ideation: a chained mediation modeling analysis
Source: Front Psychol. 2025 Nov 14;16:1658974. doi: 10.3389/fpsyg.2025.1658974 (PMC12661117; doi:10.3389/fpsyg.2025.1658974)

**Supplementary Materials1**

**Table S1 GSEM with the score of FDV as the predictive variable**

|  | *coef/se* |
| --- | --- |
| **Sleep disturbance** |  |
| FDV | 0.216*** |
|  | (0.01) |
| Age | 0.014*** |
|  | (0.00) |
| Gender | -0.526*** |
|  | (0.04) |
| Marriage | -0.588*** |
|  | (0.07) |
| Hukou | 0.272*** |
|  | (0.05) |
| Per capita monthly household income | -0.122*** |
|  | (0.03) |
| Work status | -0.147*** |
|  | (0.04) |
| Education | 0.051*** |
|  | (0.01) |
| Intercept | 5.279*** |
|  | (0.27) |
| **Anxiety** |  |
| Sleep disturbance | 0.370*** |
|  | (0.01) |
| FDV | 0.459*** |
|  | (0.01) |
| Age | -0.005** |
|  | (0.00) |
| Gender | -0.187*** |
|  | (0.05) |
| Marriage | -0.434*** |
|  | (0.08) |
| Hukou | -0.137* |
|  | (0.06) |
| Per capita monthly household income | -0.037 |
|  | (0.04) |
| Work status | -0.004 |
|  | (0.04) |
| Education | 0.016* |
|  | (0.01) |
| Intercept | 2.911*** |
|  | (0.33) |
| **SSI** |  |
| FDV | 0.149*** |
|  | (0.01) |
| Sleep disturbance | 0.00100 |
|  | (0.01) |
| Anxiety | 0.291*** |
|  | (0.01) |
| Age | 0.007*** |
|  | (0.00) |
| Gender | 0.100* |
|  | (0.04) |
| Marriage | -0.103 |
|  | (0.06) |
| Hukou | -0.0580 |
|  | (0.04) |
| Per capita monthly household income | 0.0430 |
|  | (0.03) |
| Work status | 0.00500 |
|  | (0.03) |
| Education | -0.017** |
|  | (0.01) |
| Intercept | -3.654*** |
|  | (0.24) |
| var (e. Sleep disturbance) | 10.921*** |
|  | (0.10) |
| var (e. Anxiety) | 15.789*** |
|  | (0.15) |
| N | 21916 |

Note: The coefficients under robust standard errors were all presented, with the robust standard errors shown in parentheses. ** p < 0.05, ** p < 0.01, *** p < 0.001.*

**Table S2 The results of the GSEM with different types of FDV as predictive variables**

|  | All Sample | Female | Male | Adolescent sample | Youth and middle-aged sample | Elderly sample |
| --- | --- | --- | --- | --- | --- | --- |
|  | *coef/se* | *coef/se* | *coef/se* | *coef/se* | *coef/se* | *coef/se* |
| **Sleep disturbance** |  |  |  |  |  |  |
| Sexual violence | -0.169 | -0.035 | -0.323* | 0.036 | -0.181 | -0.298 |
|  | (0.09) | (0.13) | (0.14) | (0.23) | (0.12) | (0.24) |
| Controlling violence | -0.015 | -0.153 | 0.141 | 0.290 | -0.0610 | -0.0210 |
|  | (0.08) | (0.11) | (0.12) | (0.19) | (0.10) | (0.21) |
| Emotional neglect violence | 0.677*** | 0.657*** | 0.704*** | 0.734*** | 0.766*** | 0.219 |
|  | (0.07) | (0.10) | (0.11) | (0.18) | (0.09) | (0.20) |
| Criticizing violence | 1.282*** | 1.417*** | 1.113*** | 0.982*** | 1.272*** | 1.381*** |
|  | (0.06) | (0.09) | (0.10) | (0.18) | (0.08) | (0.17) |
| Physical violence | 0.339*** | 0.270* | 0.429*** | 0.347 | 0.206 | 0.862*** |
|  | (0.09) | (0.12) | (0.13) | (0.21) | (0.11) | (0.22) |
| Age | 0.015*** | 0.016*** | 0.015*** | 0.159*** | 0.009** | 0.022** |
|  | (0.00) |  | (0.00) | (0.04) | (0.00) | (0.01) |
| Gender | -0.466*** |  |  | -0.402*** | -0.448*** | -0.377*** |
|  | (0.04) |  |  | (0.12) | (0.05) | (0.10) |
| Marriage | -0.606*** | -0.442*** | -0.758*** | -0.332 | -0.530*** | -0.562*** |
|  | (0.07) | (0.10) | (0.10) | (0.67) | (0.09) | (0.14) |
| Hukou | 0.261*** | 0.338*** | 0.180** | 0.0780 | 0.285*** | 0.245* |
|  | (0.05) | (0.07) | (0.07) | (0.12) | (0.06) | (0.11) |
| Per capita monthly household income | -0.108*** | -0.094* | -0.127** | -0.202** | -0.097** | 0.0240 |
|  | (0.03) | (0.04) | (0.04) | (0.07) | (0.04) | (0.07) |
| Work status | -0.127*** | -0.029 | -0.208*** | -0.515* | -0.0430 | 0.264 |
|  | (0.04) | (0.05) | (0.05) | (0.22) | (0.04) | (0.14) |
| Education | 0.050*** | 0.052*** | 0.049*** | 0.059* | 0.054*** | -0.022* |
|  | (0.01) | (0.01) | (0.01) | (0.03) | (0.01) | (0.01) |
| Intercept | 4.810*** | 3.774*** | 4.442*** | 4.190*** | 4.729*** | 2.969*** |
|  | (0.27) | (0.38) | (0.37) | (1.04) | (0.35) | (0.84) |
| **Anxiety** |  |  |  |  |  |  |
| Sleep disturbance | 0.378*** | 0.389*** | 0.366*** | 0.493*** | 0.378*** | 0.281*** |
|  | (0.01) | (0.01) | (0.01) | (0.02) | (0.01) | (0.02) |
| Sexual violence | 1.163*** | 0.969*** | 1.317*** | 1.201*** | 1.122*** | 1.311*** |
|  | (0.12) | (0.16) | (0.17) | (0.31) | (0.14) | (0.28) |
| Controlling violence | 0.669*** | 0.633*** | 0.691*** | 0.636* | 0.598*** | 0.947*** |
|  | (0.10) | (0.14) | (0.15) | (0.26) | (0.12) | (0.24) |
| Emotional neglect violence | 0.906*** | 1.059*** | 0.735*** | 0.899*** | 0.834*** | 1.102*** |
|  | (0.09) | (0.12) | (0.13) | (0.24) | (0.11) | (0.22) |
| Criticizing violence | 0.848*** | 0.778*** | 0.930*** | 1.120*** | 0.856*** | 0.726*** |
|  | (0.08) | (0.11) | (0.12) | (0.24) | (0.09) | (0.19) |
| Physical violence | 0.841*** | 0.688*** | 0.979*** | 0.819** | 0.933*** | 0.386 |
|  | (0.11) | (0.15) | (0.16) | (0.28) | (0.13) | (0.25) |
| Age | -0.005** | -0.010*** | -0.001 | -0.053 | -0.015*** | 0.0120 |
|  | (0.00) | (0.00) | (0.00) | (0.06) | (0.00) | (0.01) |
| Gender | -0.133* |  |  | -0.281 | -0.152* | 0.0130 |
|  | (0.05) |  |  | (0.16) | (0.07) | (0.12) |
| Marriage | -0.491*** | -0.346** | -0.630*** | 0.378 | -0.509*** | -0.172 |
|  | (0.08) | (0.12) | (0.12) | (0.91) | (0.10) | (0.16) |
| Hukou | -0.114 | -0.032 | -0.173* | 0.057 | -0.143 | 0.00600 |
|  | (0.06) | (0.08) | (0.09) | (0.17) | (0.07) | (0.13) |
| Per capita monthly household income | -0.043 | -0.104* | 0.011 | -0.195* | 0.0150 | -0.0160 |
|  | (0.04) | (0.05) | (0.05) | (0.09) | (0.04) | (0.08) |
| Work status | 0.010 | 0.042 | -0.021 | -0.750* | -0.0490 | -0.148 |
|  | (0.04) | (0.06) | (0.06) | (0.29) | (0.05) | (0.16) |
| Education | 0.021** | 0.013 | 0.026* | 0.081* | 0.0110 | 0.0120 |
|  | (0.01) | (0.01) | (0.01) | (0.04) | (0.01) | (0.01) |
| Intercept | 2.673*** | 3.020*** | 1.958*** | 5.628*** | 2.851*** | 1.539 |
|  | (0.33) | (0.46) | (0.47) | (1.41) | (0.43) | (0.95) |
| **SSI** |  |  |  |  |  |  |
| Sleep disturbance | 0.011 | 0.014 | 0.009 | 0.065*** | -0.003 | 0.0230 |
|  | (0.01) | (0.01) | (0.01) | (0.02) | (0.01) | (0.01) |
| Anxiety | 0.294*** | 0.272*** | 0.317*** | 0.276*** | 0.287*** | 0.349*** |
|  | (0.01) | (0.01) | (0.01) | (0.01) | (0.01) | (0.01) |
| Sexual violence | 1.029*** | 0.901*** | 1.166*** | 1.014*** | 1.012*** | 0.947*** |
|  | (0.07) | (0.10) | (0.11) | (0.18) | (0.09) | (0.18) |
| Controlling violence | 0.216*** | 0.256** | 0.163 | 0.131 | 0.243** | 0.150 |
|  | (0.07) | (0.09) | (0.10) | (0.16) | (0.08) | (0.16) |
| Emotional neglect violence | 0.124* | 0.076 | 0.192* | -0.0170 | 0.176* | 0.104 |
|  | (0.06) | (0.08) | (0.09) | (0.15) | (0.08) | (0.15) |
| Criticizing violence | -0.296*** | -0.217** | -0.391*** | -0.0920 | -0.325*** | -0.298* |
|  | (0.06) | (0.08) | (0.09) | (0.15) | (0.07) | (0.14) |
| Physical violence | 0.483*** | 0.488*** | 0.453*** | 0.543*** | 0.393*** | 0.696*** |
|  | (0.07) | (0.09) | (0.10) | (0.16) | (0.08) | (0.17) |
| Age | 0.006*** | 0.005** | 0.005* | -0.107** | 0.004 | -0.00200 |
|  | (0.00) | (0.00) | (0.00) | (0.04) | (0.00) | (0.01) |
| Gender | 0.079* |  |  | 0.0800 | 0.0960 | 0.0260 |
|  | (0.04) |  |  | (0.11) | (0.05) | (0.09) |
| Marriage | -0.126* | -0.063 | -0.169 | 0.724 | -0.106 | -0.171 |
|  | (0.06) | (0.08) | (0.09) | (0.62) | (0.07) | (0.12) |
| Hukou | -0.009 | -0.022 | 0.009 | 0.0140 | -0.0150 | 0.0860 |
|  | (0.04) | (0.06) | (0.06) | (0.11) | (0.05) | (0.10) |
| Per capita monthly household income | 0.027 | 0.018 | 0.031 | -0.0160 | -0.0340 | 0.202** |
|  | (0.03) | (0.04) | (0.04) | (0.06) | (0.03) | (0.06) |
| Work status | 0.005 | 0.050 | -0.032 | -0.274 | -0.099* | 0.360* |
|  | (0.03) | (0.04) | (0.04) | (0.19) | (0.04) | (0.14) |
| Education | -0.013* | -0.016* | -0.008 | 0.0290 | 0.00900 | -0.028** |
|  | (0.01) | (0.01) | (0.01) | (0.02) | (0.01) | (0.01) |
| Intercept | -3.553*** | -3.333*** | -3.549*** | -1.061 | -3.064*** | -5.373*** |
|  | (0.24) | (0.33) | (0.34) | (0.91) | (0.32) | (0.78) |
| var (e. Sleep disturbance) | 10.765*** | 10.879*** | 10.630*** | 10.963*** | 10.655*** | 10.592*** |
|  | (0.10) | (0.15) | (0.14) | (0.27) | (0.12) | (0.23) |
| var (e. Anxiety) | 16.193*** | 15.558*** | 16.789*** | 20.132*** | 15.937*** | 13.515*** |
|  | (0.15) | (0.21) | (0.23) | (0.50) | (0.19) | (0.30) |
| N | 21916 | 10958 | 10958 | 3222 | 14571 | 4123 |

Note: The coefficients under robust standard errors were all presented, with the robust standard errors shown in parentheses. ** p < 0.05, ** p < 0.01, *** p < 0.001.*

**
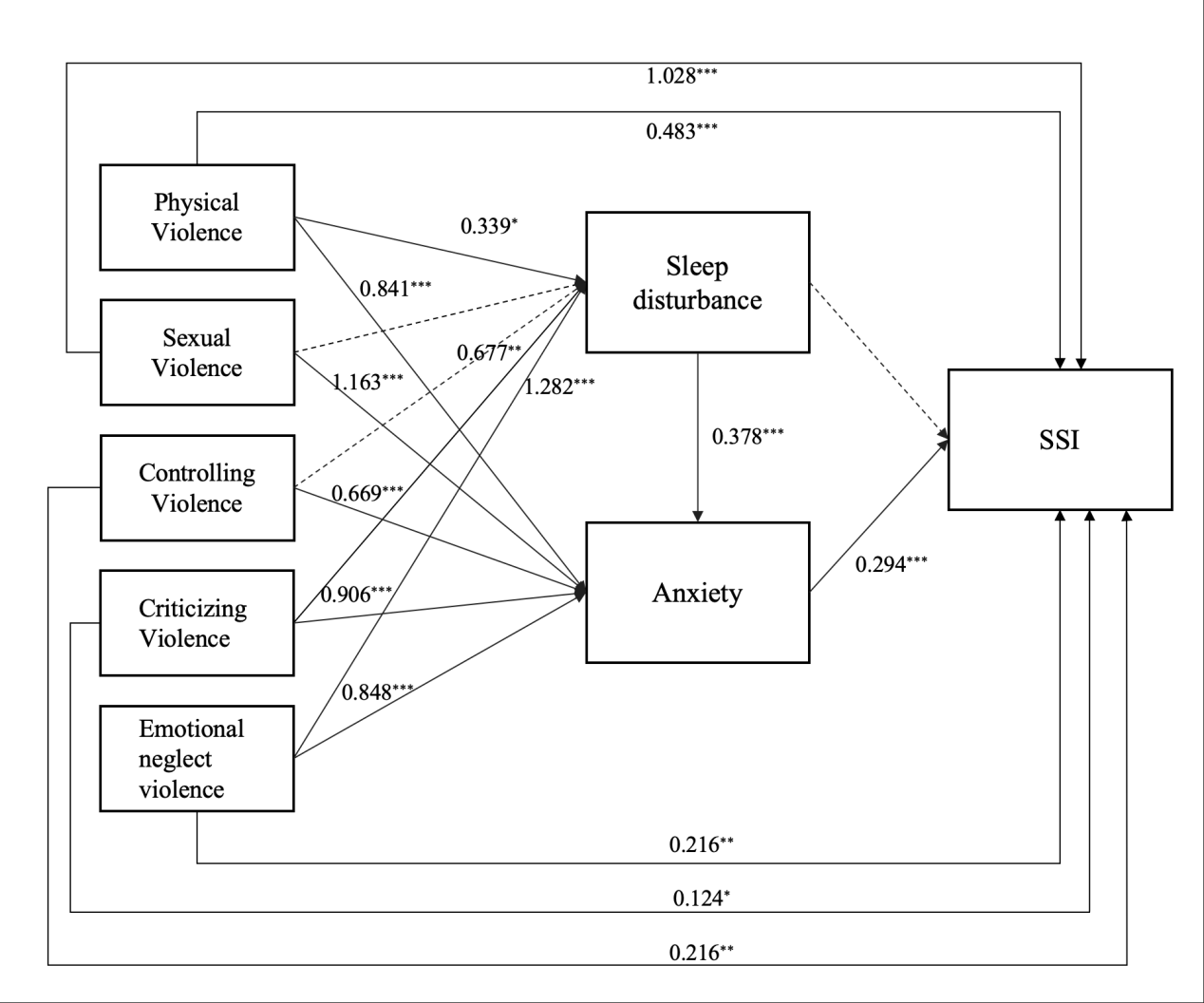
Figure S1** Chained mediation modeling of the associations among Family and Domestic Violence, Sleep Disturbance, Anxiety, and Suicidal and self-harm ideation.

Note: AUC=0.800,CFI=0.902,RMSEA=0.048. Solid lines represent that the coefficients are significant (0.05), while dashed lines indicate non-significance. Only the significant coefficients are presented in the diagram. ** p < 0.05, ** p < 0.01, *** p < 0.001*.

**
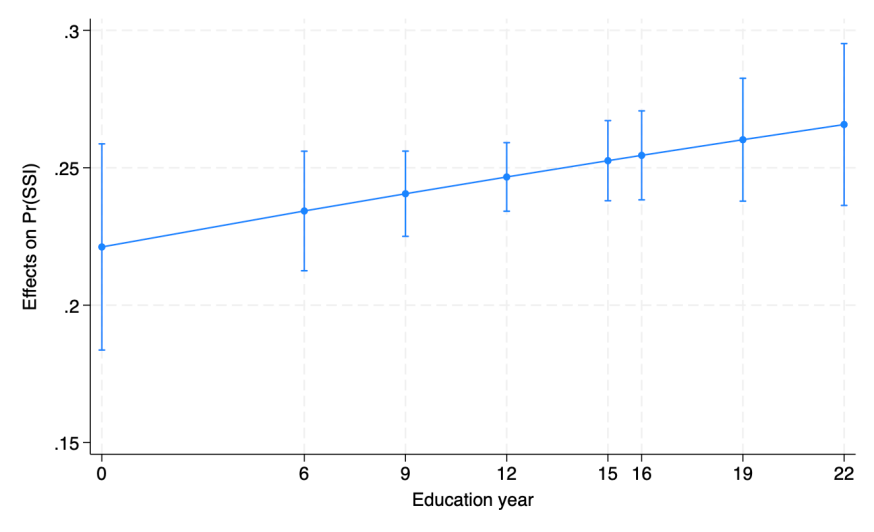
Figure S2** The marginal effect of emotional neglect violence on SSI across different educational levels


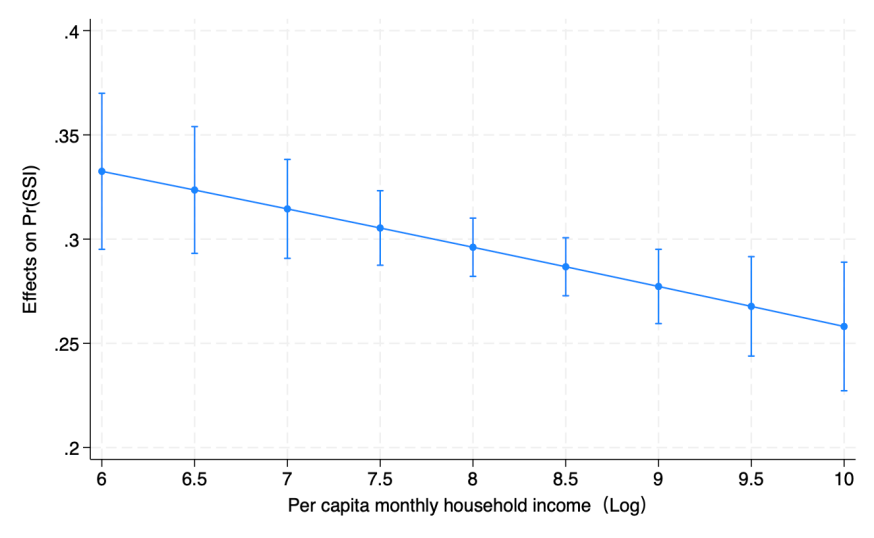
**Figure S3** The marginal effect of exposure to critical violence on SSI across different income levels

**Figure S4** The marginal effect of exposure to controlling violence on SSI across different income levels
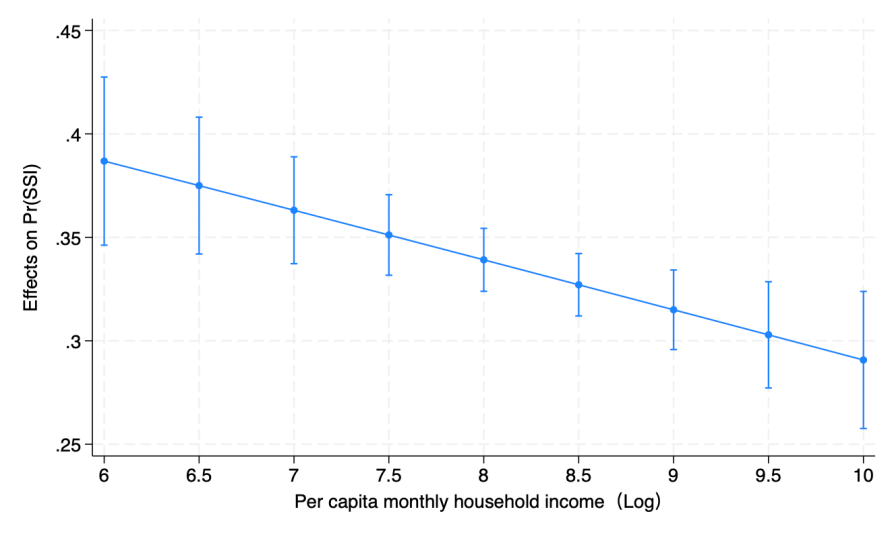

Supplement: Supplementary file 1 [file Supplementary_file_1.docx]
